# Prevalence and Clinical Impact of Incidental Extracardiovascular Findings in Pre-TAVI CT Imaging

**DOI:** 10.3390/jcm14207394

**Published:** 2025-10-20

**Authors:** Matteo Haupt, Tim Bellersen, David Weiss, Arne Bischoff, Bastian Schrader, Andreas Martens, Martin H. Maurer, Rohit Philip Thomas

**Affiliations:** 1 Department of Diagnostic and Interventional Radiology, Carl von Ossietzky Universität Oldenburg, 26192 Oldenburg, Germany; 2 University Clinic for Cardiology—Klinikum Oldenburg, Carl von Ossietzky Universität Oldenburg, 26192 Oldenburg, Germany; 3 Clinic for Cardiac Surgery, University Clinic Oldenburg, Carl von Ossietzky Universität Oldenburg, 26192 Oldenburg, Germany

**Keywords:** incidental findings, pre-TAVI CT, extracardiovascular findings, TAVI

## Abstract

**Objectives:** To evaluate the prevalence, classification, and clinical relevance of incidental extracardiovascular findings in pre-transcatheter aortic valve implantation (TAVI) CT imaging. **Methods:** We conducted a retrospective single-center study of 225 patients undergoing pre-TAVI contrast-enhanced, ECG-gated CT scans between 2021 and 2023. Extracardiovascular findings were recorded and categorized into three groups based on presumed clinical relevance: Group A (findings with no need for follow-up), Group B (findings requiring follow-up), and Group C (findings requiring immediate intervention or treatment). Statistical analysis included a descriptive assessment of the overall prevalence of incidental findings and evaluation of age- and sex-related trends using chi-square tests with Bonferroni-adjusted pairwise comparisons. **Results:** The study cohort included 225 patients (53.3% male; mean age 79.9 ± 6.2 years, range 58–93). Extracardiovascular incidental findings were detected in 205 patients (91.1%). Among all 478 recorded findings, 82.6% were Group A, 14.4% Group B, and 2.9% Group C. On a per-patient level, 87.1% had at least one Group A finding, 24.9% had at least one Group B, and 6.2% had at least one Group C finding. Older age was associated with more incidental findings, with a significant difference observed between the 70–79 and 80–89 age groups (*p* = 0.002). No significant sex-related differences were found (*p* = 0.226). Findings were most frequently located in the abdomen (46.2%) and thorax (37.2%). Among all clinically relevant findings, the thorax was the most commonly affected region: 43.5% of Group B and 78.6% of Group C findings were located in the thorax, followed by the abdomen (33.3% of Group B and 7.1% of Group C findings). **Conclusions:** Extracardiovascular incidental findings are highly prevalent in pre-TAVI CT imaging and range from benign, age-related changes to potentially serious conditions such as malignancies or infections. Their presence reflects the comorbidity burden of the typical TAVI population and underscores the importance of recognizing non-vascular incidental findings in this clinical setting.

## 1. Introduction

Transcatheter aortic valve implantation (TAVI) has become an established treatment for severe aortic valve stenosis, particularly in elderly patients at elevated surgical risk [1]. Pre-procedural CT imaging is now a cornerstone of TAVI planning, providing detailed information on aortic root anatomy and vascular access routes [2]. Because the standard TAVI CT protocol typically includes parts of the thorax, abdomen, and pelvis, it covers a relatively wide anatomical range in which extracardiovascular incidental findings are frequently encountered. These findings are unrelated to the cardiac valve pathology but often reflect the burden of comorbidities in an elderly population.

Such incidental findings can pose a clinical dilemma: while most are benign or of limited immediate relevance, some may represent serious conditions (e.g., malignancies or infections) that could influence the timing or feasibility of TAVI.

Several previous studies have examined the prevalence of incidental findings on pre-TAVI imaging. Reported rates vary widely—from approximately 57% of patients up to nearly 99%—depending on how “incidental finding” is defined and which thresholds are applied [3,4]. This variation is also partly due to the lack of a standardized classification system across studies. Some authors use a binary classification, distinguishing between “clinically significant” and “not significant” based on whether further clinical action is required. Others have introduced a three-tier system that stratifies incidental findings by clinical relevance: insignificant (no action needed), intermediate (requiring follow-up or work-up but with no immediate impact on the TAVI procedure), and significant (requiring urgent evaluation or possibly representing a contraindication to TAVI) [4,5].

Despite these differences in classification, there is broad agreement in the literature that extracardiovascular incidental findings are common in this patient population and may, in some cases, have important clinical implications.

Given this context, we aimed to systematically assess the prevalence and types of incidental extracardiovascular findings in our cohort of patients undergoing CT for TAVI planning. We also sought to apply a structured classification system (Groups A, B, and C as defined below) to these findings and to evaluate their clinical relevance. Additionally, we examined whether certain patient characteristics—such as age or sex—are associated with a higher likelihood of significant incidental findings. By following this approach, we emphasize the importance of structured detection and evaluation of such findings, which can support interdisciplinary collaboration and minimize disruptions to the TAVI workflow.

## 2. Methods

**Study Design and Population:** We conducted a retrospective, single-center cohort study, reviewing the records of 225 consecutive patients who underwent pre-TAVI CT imaging at the Klinikum Oldenburg, Germany, between 2021 and 2023. All patients had severe symptomatic aortic stenosis and were evaluated by the heart team for TAVI. The CT scans were performed as part of the standard pre-TAVI work-up during the study period.

**CT Imaging Protocol:** All patients underwent contrast-enhanced, ECG-gated multidetector CT (MDCT) of the heart and a contrast-enhanced aorto-ilio-femoral run-off from the lower abdomen to the upper thighs for arterial vessel visualization. This protocol covered most of the abdominal and the iliofemoral arteries, as well as partially encompassing the thoracic and abdominal organs. The resulting images were assessed on both dedicated cardiac reconstructions and wider field-of-view images to ensure that extracardiovascular structures were adequately evaluated.

**Incidental Findings Classification:** At least two experienced radiologists reviewed each CT. Each incidental finding was recorded and categorized into one of three groups based on presumed clinical relevance:

1. **Group A (Findings with no need for follow-up):** Incidental abnormalities considered benign or clinically no need for follow-up. These required no further diagnostic work-up or intervention. Examples include uncomplicated diverticulosis or simple renal cysts not requiring further evaluation.
2. **Group B (Findings requiring follow-up):** Incidental findings that were not immediately life-threatening or procedure-altering, but did warrant additional evaluation or surveillance. This category includes lesions of uncertain significance that might need follow-up imaging or specialist consultation (for instance, a solid renal mass suggestive of a renal tumor, a lung nodule above a certain size threshold, enlarged lymph nodes, etc.). These findings had potential clinical importance but did not necessarily preclude or delay TAVI unless the work-up indicated otherwise.
3. **Group C (Findings requiring immediate intervention or treatment):** Incidental findings of a potentially serious nature that could significantly impact patient management or the TAVI procedure timeline. This includes conditions requiring urgent treatment or posing a contraindication to immediate TAVI. Examples might be an active infection (e.g., pneumonia on imaging requiring prompt therapy) or a newly discovered malignancy that demands intervention. Group C findings were those likely to prompt changes in the planned approach or timing of TAVI (such as postponement or additional pre-TAVI treatments).

This classification is based on systems used in previous studies, where incidental findings are categorized according to whether they require no action, further (but not urgent) action, or urgent action (e.g., [6]). In cases where multiple incidental findings were present in a single patient, each finding was classified individually; the patient could therefore have findings in more than one category; for example, a patient with both a benign cyst and a suspicious lung lesion would be noted in both Group A and B counts. For pulmonary nodules, we applied the Fleischner Society guidelines [7]: nodules smaller than 6 mm in low-risk patients were classified as Group A, whereas larger or morphologically suspicious nodules were assigned to Group B.

**Data Collection:** For each patient, demographic data, including age and sex, were collected. All extracardiovascular findings detected on pre-TAVI CT scans were systematically documented and categorized into Groups A, B, or C based on clinical relevance. Each finding was also classified based on its anatomical location, distinguishing between the thorax, abdomen, pelvis, and musculoskeletal system.

**Statistical Analysis:** We first calculated the prevalence of incidental findings overall and by category. The continuous variable age was summarized as mean ± standard deviation (SD). Categorical variables were compared using chi-square tests. We specifically analyzed the distribution of incidental findings across different age groups, given an a priori hypothesis that older patients may have more comorbid findings. A chi-square test was also used to assess differences by sex. A *p*-value of <0.05 was considered statistically significant; for post hoc tests, a Bonferroni-corrected threshold of *p* < 0.017 was applied.

All statistical analyses were conducted using the software JASP, Version 0.19.1 (University of Amsterdam, Amsterdam, The Netherlands). The study was approved by the institutional ethics committee.

## 3. Results

### 3.1. Patient Demographics

The study included 225 patients (53.3% male, 46.7% female). The mean age was 79.9 ± 6.2 years (median 81, interquartile range 76–84, range 58–93 years), reflecting the typical elderly TAVI population. Key baseline characteristics are summarized in Table 1. Notably, over 80% of the cohort was over the age of 70, and around 60% were over 80 years old, consistent with an age group in which incidental findings are expected to be common.

### 3.2. Prevalence of Incidental Findings

A vast majority of patients had at least one incidental extracardiovascular finding on their pre-TAVI CT. An overview is provided in Table 2. In total, 205 out of 225 patients (91.1%) had one or more incidental findings beyond the cardiac and vascular system, while only 8.9% of patients had a completely normal extracardiovascular scan (Table 2). Across all patients, a total of 478 individual incidental findings were identified, of which 395 (82.6%) were classified as Group A, 69 (14.4%) as Group B, and 14 (2.9%) as Group C.

On a per-patient basis, 87.1% of patients (196/225) had at least one Group A finding, while 24.9% (56/225) had at least one Group B finding and 6.2% (14/225) had at least one Group C finding. Many patients had multiple incidental findings; in fact, 51.6% (116/225) had two or more extracardiovascular abnormalities documented. Among patients with any incidental finding, the median number of findings was 2 (range 1–6).

**Figure 1:**
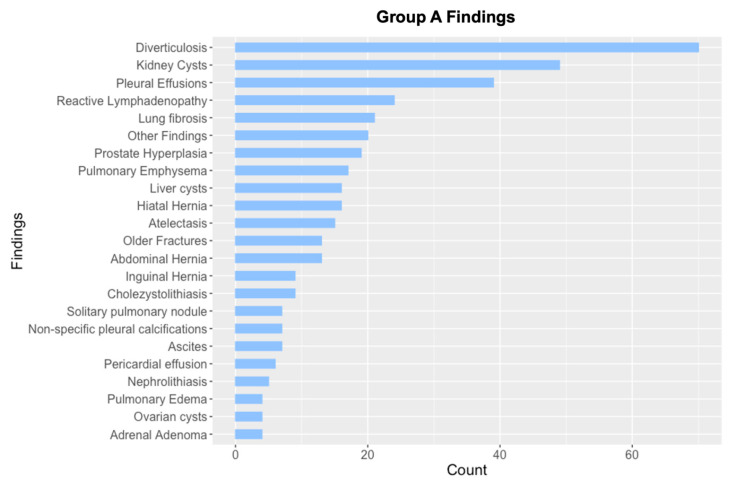
Overview of Group A findings (Findings with no need for follow-up), sorted by count. Findings with n ≤ 3 are grouped under “Other findings”. Total number of Group A findings: n = 395.

**Figure 2:**
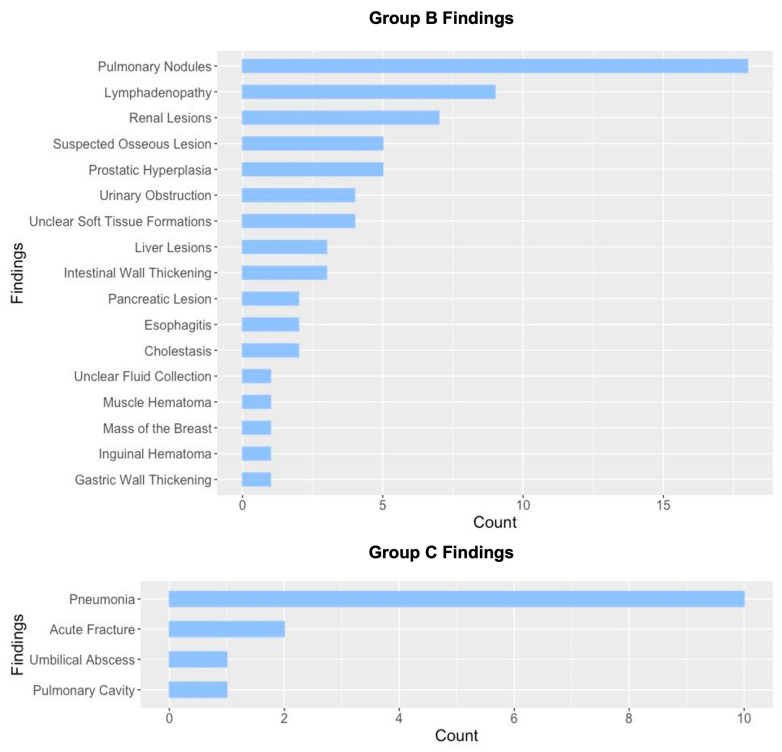
Overview of Group B (findings requiring follow-up) and Group C (findings requiring immediate intervention or treatment), sorted by count. Total number of Group B findings: n = 69; total number of Group C findings: n = 14.

The most frequently observed Group A findings included diverticulosis and simple renal cysts (Figure 1). In contrast, the most common Group B findings consisted of lung nodules requiring follow-up or histopathological evaluation, adrenal masses, and renal lesions suspicious for malignancy (Figure 2). Group C findings were relatively rare but clinically significant, with pneumonia (10 cases, 71.4% of Group C findings) being the most frequently observed critical finding, followed by acute fractures (2 cases, 14.3%) (Figure 2). Additionally, a pulmonary cavity with suspected tuberculosis and an umbilical abscess were detected. In patients with category C findings, TAVI was typically postponed until the acute condition (e.g., pneumonia, infection, or fracture) had resolved. This represents an important clinical aspect, as such findings may directly influence procedural timing.

**Figure 3:**
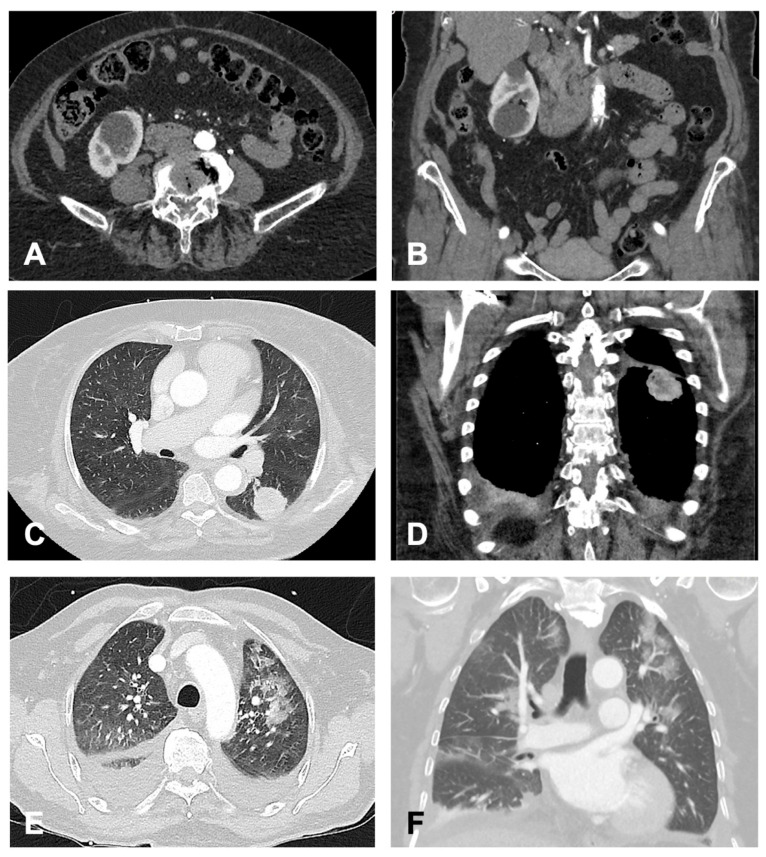
Exemplary Findings of Categories (**A**–**C**), Each Shown in Transverse and Coronal Planes. (**A**,**B**) (Group A): 82-year-old female patient with incidental right-sided renal cysts. (**C**,**D**) (Group B): 74-year-old female patient with an incidentally detected mass in the apical segment of the left lower lobe. Subsequent histopathological examination confirmed an adenocarcinoma. (**E**,**F**) (Group C): 72-year-old male patient with detected bilateral pneumonic infiltrates. Additionally, bilateral pleural effusions with adjacent ventilation impairment were observed. Given the suspicion of nosocomial pneumonia, antibiotic therapy was initiated.

Additionally, Figure 3 provides representative CT images for selected findings from each category, highlighting examples of benign, indeterminate, and clinically significant incidental findings.

### 3.3. Age- and Sex-Related Trends

**Figure 4:**
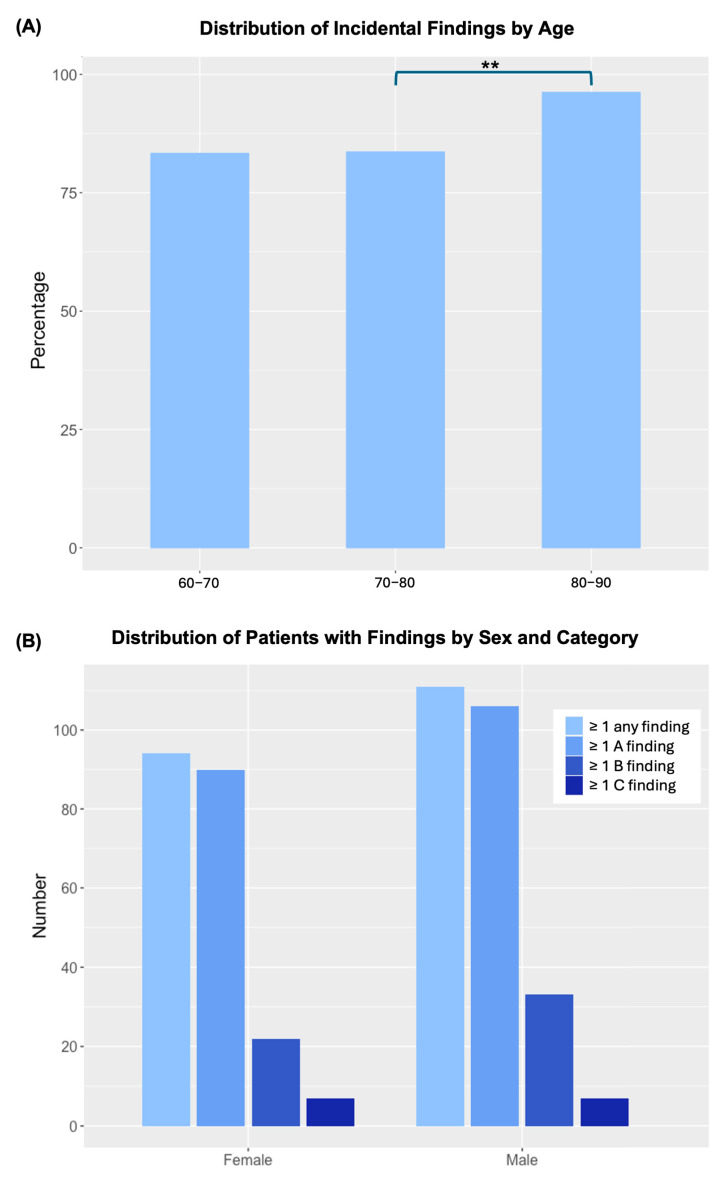
Distribution of incidental findings by age, category, and sex. (**A**) Percentage of patients with at least one incidental finding across three age groups ([60, 70), [70, 80), and [80, 90)). Bars with ** indicate significant differences between groups (*p* < 0.017, Bonferroni-adjusted). Age groups < 60 years (n = 1) and ≥90 years (n = 7) are shown descriptively only (no statistical testing due to small sample sizes). (**B**) Distribution by sex and finding category. No significant association between sex and the presence of incidental findings was observed.

Incidental findings were analyzed across age groups. Figure 4 shows the percentage of patients with incidental findings stratified by age. Patients in the 80–89 age group had the highest prevalence, with 96.2% having at least one extracardiovascular finding on CT. In comparison, the prevalence was 83.6% in the 70–79 age group and 83.0% in the 60–69 age group.

Statistical analysis using a chi-squared test revealed a significant association between age group and the presence of incidental findings (χ^2^(2) = 10.41, *p* = 0.005). Post hoc comparisons showed that participants aged 80–89 years had a significantly higher rate of incidental findings than those aged 60–69 years (χ^2^(1) = 3.95, *p* = 0.047) and 70–79 years (χ^2^(1) = 9.89, *p* = 0.002). There was no significant difference between the 60–69 and 70–79 age groups (χ^2^(1) = 0.0004, *p* = 0.984). After applying a Bonferroni correction (*p* < 0.017), only the comparison between the 70–79 and 80–89 age groups remained statistically significant. Patients younger than 60 years (n = 1) and those aged ≥90 years (n = 7) were excluded from the age-group comparison due to small sample sizes. These patients were, however, included in all overall analyses and descriptive summaries.

A chi-squared test revealed no statistically significant association between sex and the presence of incidental findings (χ^2^(1) = 1.46, *p* = 0.226; Figure 4). However, certain types of findings were obviously sex-specific, with prostate lesions detected exclusively in men and ovarian cysts in women.

### 3.4. Types and Anatomical Distribution

**Figure 5:**
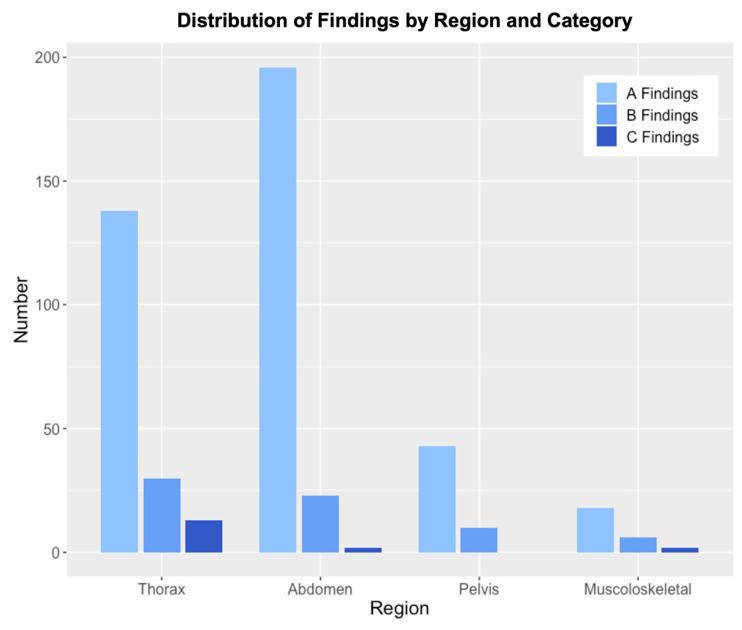
Distribution of Findings by Anatomic Region and Group. The figure illustrates the number of findings across four anatomical regions (Thorax, Abdomen, Pelvis, and Musculoskeletal). Each region is represented by grouped bars, categorized into A Findings (Findings with no need for follow-up), B Findings (Findings requiring follow-up), and C Findings (Findings requiring immediate intervention or treatment).

Incidental findings were most frequently located in the thorax and abdomen, which together accounted for the majority of observations (Figure 5). A total of 178 thoracic findings were identified, including pulmonary nodules and fibrosis, as well as thyroid lesions. Similarly, abdominal findings were highly prevalent, with 221 cases involving renal cysts or masses, adrenal nodules, liver lesions (such as cysts or hemangiomas), gallstones, pancreatic cysts, and colonic diverticulosis. Findings in the pelvis and musculoskeletal system were less frequent, with 53 pelvic findings primarily consisting of ovarian cysts and bladder diverticula, and 26 musculoskeletal findings, including vertebral compression fractures and incidental bone lesions.

Thoracic and abdominal findings contributed most to the Group A and B categories. The musculoskeletal and pelvic findings were largely benign and less frequently observed. In contrast, Group C findings were relatively rare, with most occurring in the thorax. Pneumonia was the most frequently observed critical finding, followed by acute fractures, while a pulmonary cavity with suspected tuberculosis and an umbilical abscess were also detected. The majority of Group C findings (78.6%) were thoracic; the remainder occurred in the musculoskeletal system (14.3%) and abdomen (7.1%).

## 4. Discussion

Our study confirms that incidental extracardiovascular findings are exceedingly common in patients undergoing pre-TAVI CT imaging. We observed incidental findings in approximately 91% of our cohort, which is consistent with the high rates reported in other studies [6,8]. In the literature, reported prevalence ranges from just over 50% in more narrowly defined cohorts to over 95% [8,9]. This wide variation is largely due to differences in definitions, patient populations, and imaging protocols. Some early studies only included clinically significant findings, whereas others—like ours—documented all findings regardless of immediate clinical impact. Our 91% prevalence likely reflects this comprehensive documentation and highlights the reality that an elderly TAVI population nearly always presents with coexisting pathologies visible on CT.

By applying a structured classification (Groups A, B, C), we were able to stratify these findings by clinical relevance. This approach is analogous to classifications used in previous studies [6]. In our cohort, approximately 17% of all findings (Groups B + C) were of potential clinical concern, either requiring follow-up or urgent action. Consistent with these findings, Staab et al. found that 17.1% of their TAVI patients had clinically significant extracardiovascular findings [10]. Hinton et al. (2019) reported about 14% of patients with at least one significant incidental finding in a large UK cohort [6]. Our results show that approximately 30% of patients had at least one incidental finding requiring additional evaluation, with 24.9% classified as Group B and 6.2% as Group C. This proportion is slightly higher than in some previous studies, possibly due to our broader definition of Group B follow-up findings. However, our findings remain consistent with prior research, which indicates that one-quarter to one-third of TAVI patients present with incidental findings that necessitate attention beyond the TAVI procedure.

The anatomical distribution of findings in our study showed that the thorax and abdomen were the most frequently affected regions. This is expected given the scan coverage and the high prevalence of chronic conditions in the elderly TAVI population. Most findings were located in these two regions, while pelvic and musculoskeletal findings were less common. The thorax mainly included pulmonary nodules, pleural effusions, and mediastinal abnormalities. Abdominal findings were largely renal, hepatic, adrenal, and gastrointestinal lesions. Pelvic findings consisted primarily of ovarian cysts and bladder diverticula; musculoskeletal findings were mostly vertebral changes and incidental bone lesions. This distribution aligns with previous studies, such as Fathala et al., who reported incidental abnormalities in 85% of abdominal scans and 69% of thoracic scans [11].

Common chest findings such as pulmonary nodules, fibrotic changes, and pleural effusions were frequently observed. Given that TAVI candidates often present with advanced cardiac disease and associated comorbidities, the presence of pleural effusions is not unexpected and may reflect underlying congestive heart failure or volume overload. The high incidence of small pulmonary nodules, however, remains a diagnostic challenge, particularly in differentiating lesions that warrant further investigation from those that can be safely monitored without delaying the TAVI procedure. Many nodules represent incidental granulomas, but some may require additional work-up, such as follow-up imaging, PET/CT, or biopsy.

Abdominal findings in our patients were often benign, including cysts, gallstones, and diverticulosis. However, some required further evaluation, such as renal or pancreatic lesions. This underscores the importance of a thorough review, as relevant pathologies—such as early-stage malignancies—might otherwise go undetected until progression.

The prognostic implications of incidental findings remain debated. It is plausible that potentially malignant findings negatively affect survival, either due to the underlying disease or because further work-up and treatment contribute to a worse overall prognosis. Refai et al. reported higher long-term mortality in patients with incidental findings (49.4% vs. 37.5%) [12]. Similarly, van Kesteren et al. found that potentially malignant incidental findings—identified in approximately 20% of patients—were independent predictors of worse five-year survival after TAVI [9]. These findings suggest that extracardiovascular pathology may influence long-term outcomes by indicating an increased disease burden or delaying treatment.

In contrast, other studies have not observed such associations. Ha et al. (2020) reported no difference in one-year mortality between patients with and without incidental findings [13]. Similarly, Stachon et al. (2017) found no impact of potentially malignant findings on heart team decision-making or two-year survival [14]. These discrepancies may reflect differences in populations, follow-up durations, and definitions. Additionally, in elderly, multimorbid patients, oncologic diagnoses may not necessarily result in active interventions, which could attenuate the impact on survival.

We also observed an age-related difference in the prevalence of incidental findings, with the highest rates among patients in their 80s—a relationship that has rarely been described. Staab et al. specifically noted a significant increase in incidental findings with advancing age [10]. This is intuitive but important to recognize. As TAVI is increasingly offered to younger and lower-risk patients, the overall rate of incidental findings may decrease slightly. However, in the classical TAVI demographic, we must continue to expect and manage a high number of incidental findings.

Another important aspect is the lack of a standardized definition for incidental findings across studies. In this context, our classification into Groups A, B, and C offers a practical framework: Group A includes findings that do not require further workup; Group B encompasses findings that warrant follow-up; and Group C includes findings that demand urgent attention. This categorization helps to prioritize care and assess the clinical relevance of extracardiovascular incidental findings in a structured manner.

This retrospective, single-center design represents a key limitation of the study and may limit the generalizability of our findings to other institutions with different referral patterns or imaging protocols. As this was a single-center study, a potential referral bias cannot be excluded, since patient characteristics and referral indications may differ from other institutions. The sample size of 225 patients is moderate and may limit generalizability. However, our results are largely in line with the existing literature. All imaging studies were initially reviewed by at least two experienced radiologists. While this approach minimized the risk of overlooked findings, some degree of inter-reader variability cannot be excluded.

Long-term follow-up or outcome data were not available, precluding an assessment of how incidental findings may have influenced patient management or prognosis. Future research should explore whether specific types of incidental findings are associated with delays, cancellations, or worse clinical outcomes. Additionally, it remains to be investigated how frequently incidental findings influence the decision-making process regarding TAVI eligibility and timing.

## 5. Conclusions

Incidental extracardiovascular findings are common in patients undergoing pre-TAVI CT due to the broad anatomical coverage of the scan and the advanced age of this population. In our study, over 90% of patients had at least one incidental finding, and about one-third required further evaluation or clinical action. The prevalence of incidental findings increased with age, with the highest rate observed in patients over 80 years. Most findings did not require further diagnostic work-up, such as diverticulosis and renal cysts. However, some patients presented with clinically significant conditions, including occult malignancies or infections.

As TAVI expands to younger, lower-risk patients, the overall burden of incidental findings may decrease. However, in the typical TAVI population, where incidental pathologies are frequent, standardized guidelines for their management will be essential.

Future studies should investigate the impact of incidental findings on TAVI decision-making and patient outcomes. Key questions include whether certain findings affect procedural eligibility, alter management strategies, or influence survival. Prospective research is needed to establish evidence-based recommendations for handling these findings in clinical practice. Until such guidelines are available, a structured and multidisciplinary approach remains crucial to ensure appropriate management without unnecessary delays in valve intervention.

## Figures and Tables

**Table 1:** Demographic Characteristics of the Study Population.

Characteristic	Value
Total Patients	225
Sex	
Male	120 (53.3%)
Female	105 (46.7%)
Age (years)	
Mean ± SD	79.9 ± 6.2
Median (IQR)	81 (76–84)
Range	58–93

**Table 2:** Extracardiovascular findings were categorized into three groups: Group A, findings with no need for follow-up; Group B, findings requiring follow-up; and Group C, findings requiring immediate intervention or treatment.

	Number	Percentage (%)
All patients	225	100
Patients without any extracardiovascular findings	20	8.9
Patients with ≥1 extracardiovascular finding	205	91.1
Patients with ≥1 Group A finding	196	87.1
Patients with ≥1 Group B finding	56	24.9
Patients with ≥1 Group C finding	14	6.2
All findings	478	100
Group A	395	82.6
Group B	69	14.4
Group C	14	2.9

## Data Availability

The data presented in this study are available on reasonable request from the corresponding author. The data are not publicly available due to privacy and ethical restrictions in accordance with institutional regulations.
